# Viral Traits and Cellular Knock-Out Genotype Affect Dependence of BVDV on Bovine CD46

**DOI:** 10.3390/pathogens10121620

**Published:** 2021-12-14

**Authors:** Hann-Wei Chen, Verena Huber, Kati Szakmary-Braendle, Kerstin Seitz, Marlene Moetz, Till Ruemenapf, Christiane Riedel

**Affiliations:** Institute of Virology, Department of Pathobiology, University of Veterinary Medicine, 1210 Vienna, Austria; Hann-Wei.Chen@vetmeduni.ac.at (H.-W.C.); 01420513@students.vetmeduni.ac.at (V.H.); Kati.Szakmary-Braendle@vetmeduni.ac.at (K.S.-B.); Kerstin.seitz@vetmeduni.ac.at (K.S.); marlene.moetz@vetmeduni.ac.at (M.M.); till.ruemenapf@vetmeduni.ac.at (T.R.)

**Keywords:** bovine viral diarrhea virus, pestivirus, bovine CD46, susceptibility, CRISPR-CAS9 mediated knock-out

## Abstract

The role of bovine CD46 in the host cell entry of BVDV has been established for more than a decade. By generating novel MDBK CD46 knock-out clones, we confirm previously reported data on the CD46 motives important for BVDV binding and the importance of the G479R exchange within BVDV E^rns^ to gain independence of bovine CD46 during entry. The comparison of different knock-out genotypes revealed a high variability of cellular susceptibility for a BVDV encoding the G479R exchange. These data highlight the effect of clonal selection of knock-outs on virus susceptibility, which should be considered when planning knock-out experiments.

## 1. Introduction

Bovine viral diarrhea virus (BVDV, species *Pestivirus A + B*) is a small, enveloped, positive-sense, single-stranded RNA virus within the genus *Pestivirus* [1] of the family *Flaviviridae*. It causes the economically important cattle disease bovine viral diarrhea and is a notifiable pathogen in many countries. A special trait of BVDV is the ability to cause persistent infection accompanied by immunotolerance in animals infected in utero. These animals are of great epidemiological importance, as they constantly shed high amounts of virus.

Pestivirus particles integrate three surface glycoproteins into their envelope [2]. E^rns^ is a dimer [3], has ribonuclease activity [4], and possesses an atypical, amphipathic membrane anchor [5]. It is essential for pestivirus infectivity, with the exception of Bungowannah virus [6]. The majority of E1 and E2 proteins are present in the virus particle as heterodimers. E2 is associated with receptor interaction, whilst the function of E1 remains unknown. 

Virus attachment and entry are essential steps in the viral replication cycle in which BVDV heavily relies on the host cell machinery to reach a cellular compartment compatible with virus fusion. During attachment, BVDV interaction with the host cell is mediated by surface glycosaminoglycans [7] and bovine CD46 [8]. Subsequently, the virus is internalized by clathrin-mediated endocytosis [9]. Interestingly, the importance of porcine CD46 for the entry of the distantly related atypical porcine pestivirus (APPV) was recently demonstrated [10]. Bovine CD46 or complement control protein is a type I transmembrane protein, whose N-terminus consists of four complement control protein (CCP) domains. CCP1—the domain most distant from the membrane—has been determined as the site of BVDV–CD46 interaction [11]. Specifically, the motives EQIV (position 66–69) and GQVLAL (position 82–87), localized on antiparallel beta sheets, are mediating binding.

The essential function of bovine CD46 as a cellular receptor has been demonstrated by employing anti-CD46 monoclonal antibodies or polyclonal sera [8,11,12], heterologous expression in porcine cells [8,11] and CRISPR/CAS9-mediated knock-out [13]. It seems to be important only for virus uptake from the supernatant, whilst cell-to-cell transmission [14] and infection of polarized alveolar epithelial cells from the basal membrane [15] are independent of CD46. During the entry process, expression of bovine CD46 in porcine cells does not affect BVDV particle surface motility, but rather increases the efficiency of virus uptake [16].

Directed knock-out of cellular genes by CRISPR/CAS9 approaches has greatly ameliorated the ease of knock-out generation [17]. However, the geno- and phenotype of such knock-out clones are not only determined by the targeted modification of the gene of interest, but also by additional factors such as off-target effects and clonal selection, which are difficult to evaluate and control [18]. Therefore, knock-out effects are ideally characterized in several clones to even out unspecific effects. The random modifications induced by a single guide RNA make screening and characterization of the resulting clones more tedious. Yet, this more random approach has the advantage of providing clones with different modifications in the gene of interest, including deletions that are preserving the reading frame.

In the presented study, we sought to determine the importance of bovine CD46 for three different BVDV strains, NADL (BVDV-1a) [19], CP7 (BVDV-1b) [19], and C87 (BVDV-1a), and the effect of the clonal selection and knock-out genotype of the CD46 knock-out cells on their susceptibility. NADL and C87 have previously been employed to study the importance of bovine CD46 during entry (see, for example, [13,14,15,16]). CP7 was included in this study as a representative of BVDV-1b strains.

## 2. Results

**Figure 1:**
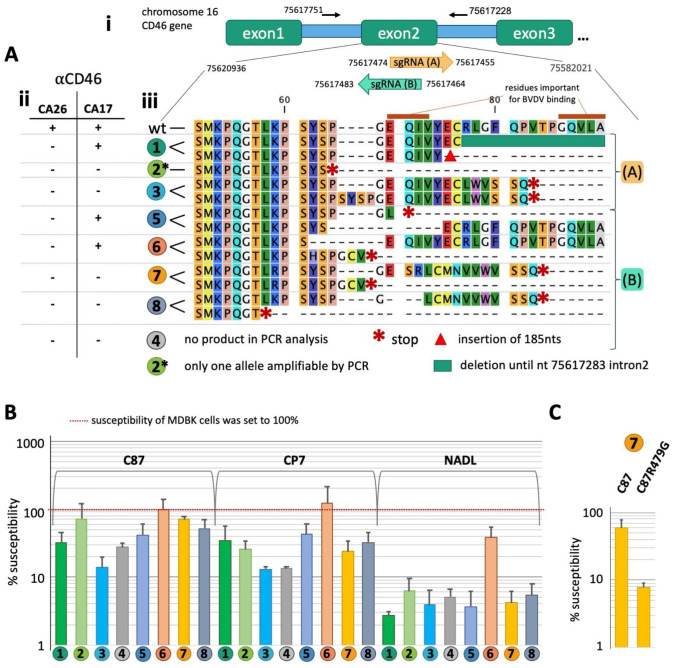
Genotype of generated MDBK CD46 knock-out cell lines and susceptibility to different BVDV-1 strains. (**A**) (**i**) Localization of the sgRNAs and the primers used for amplification of the modified region by PCR on the CD46 gene. (**ii**) Reactivity of the cell lines with anti-CD46 antibodies CA17 and CA26. − indicates no reactivity, + indicates reactivity. (**iii**) Effect of modifications within the CD46 alleles on the expressed protein. Stop codons are indicated by red asterisk. Residues important for BVDV E2 binding according to [11] are indicated. Wt = unmodified MDBK cells. (**B**) Susceptibility of the different clones to BVDV-1 strains C87, CP7, and NADL in comparison to the parental MDBK cell line. The average and standard deviation of three independent experiments are depicted. Color code and clone numbering is according to (**A**-**iii**). (**C**) Effect of the susceptibility of clone 7 to C87 and C87 encoding a G at position 479 of the polyprotein (C87R479G).

Bovine CD46 knock-out cells were generated by transduction with lentiviral vectors encoding the sgRNAs (A) or (B) (Figure 1A-i) under the control of a U6 promoter and CAS9. Two different sgRNAs were chosen in this experimental setup to account for potential differences in efficiency and to slightly vary the site of the gDNA strand break. Clones not interacting with anti-bovine CD46 monoclonal antibody CA26 [12] were clonally selected three more times. Subsequently, a final characterization with CA26 and an additional anti-bovine CD46 antibody, CA17 [12], was performed by immunofluorescence to check for the presence of CD46 on the cell surface. CA17 was employed as it was reported to bind in the CCP2 domain [11], whilst the binding site of CA26 was mapped to CCP1. Out of the eight clones in the final selection, three (1–3) were expressing sgRNA(A), whilst five (4–8) were expressing sgRNA(B). Clones 1 and 5 still showed a weak signal when detecting CD46 with CA17, whilst the CD46 surface staining of clone 6 when incubated with CA17 was comparable to the intensity observed on nonmodified MDBK cells. All other clones did not interact with CA17 (Figure 1A-ii and Supplementary Figure S1).

To determine the genotype of the eight clones, bovine CD46 exon 2 was amplified from gDNA by PCR. Sequence traces derived from the PCR product were compared with the single trace obtained from a cloned PCR fragment to determine the modification of each allele. Clone 1 encodes a deletion reaching into intron 2 on one allele, whilst the other allele contains an insertion of 185 nts (Figure 1A-iii). In clone 2, one allele lacks 21 nts and encodes one nucleotide exchange, resulting in a stop codon. Only one sequence trace could be detected in the PCR amplicon of clone 2, indicating a larger deletion preventing amplification of the other allele by PCR. One nucleotide is deleted in both alleles of clone 3, with one allele also showing a duplication of 12 nts. No PCR product could be obtained for clone 4, suggesting the presence of a deletion extending the range of the PCR in both alleles. Four nts are exchanged in one allele of clone 5, resulting in a stop codon. The other allele misses 21 nts. Twelve and eight nts are deleted in the CD46 alleles of clone 6. Clones 7 and 8 show deletions (clone 7: two and eight nts; clone 8: 38 and seven nts) changing the reading frame in both alleles, resulting in a premature stop codon.

By comparing the genotype of the CD46 knock-out clones and their interaction with the anti-CD46 antibodies CA17 and CA26, residues in CD46 affecting antibody binding could be mapped. For CA26, binding is not observed in clone 6, even though only four amino acids (62-YSPG) are deleted in one allele. The motive YSPG starting at position 62 is therefore essential for antibody binding. For CA17, staining with an intensity comparable to nonmodified cells is only observed for clone 6. Clones 1 and 5 show a reduced staining pattern, indicating the importance of amino acids 66–72 for high binding affinity.

To assess the impact of the CD46 modifications/knock-out on the susceptibility to different BVDV-1 strains, the different cell lines and the parental MDBK cell line were infected with C87, CP7, or NADL in ten-fold dilutions and the titer was determined in focus-forming units by fluorescence microscopy 48 h after infection. Susceptibility in percent of the parental MDBK cell line was calculated by dividing the virus titer obtained on a knock-out clone by the titer determined for the parental MDBK cell line (Figure 1B). For strain NADL, most modifications of CD46 and its knock-out reduced the susceptibility by 93.8–97.3%. The susceptibility of clone 6 was only reduced by 62%, indicating a residual function of CD46. Based on the genotype of the cell lines, the importance of the 66-EQIV motive for the interaction of NADL and CD46 could be confirmed. The susceptibility to strains C87 (*p* < 0.003) and CP7 (*p* < 0.05) was significantly different from the susceptibility to strain NADL. In case of double knock-out, susceptibility to C87 was reduced 28–86% and to CP7 67–87%. The four-amino-acid deletion of clone 6 did not result in a reduced susceptibility for both C87 and CP7. Interestingly, the susceptibility of clones 1 and 5 to infection with C87 or NADL was lower than observed with the double knock-out clones 2 and 7. Together with the variable effect of knock-out on susceptibility (range for C87 14–72%, for NADL 3.9–6.2%), it is likely that the phenotype of the knock-out clones is substantially affected by clone-specific factors other than the changes in the CD46 gene.

The three strains employed in this experiment differed in the E^rns^ protein at position G479 of the polyprotein. The exchange of this residue to an arginine (R479) has previously been reported to affect the in vitro dependence of BVDV on CD46 [13]. The sequence of the full-length clones of NADL and CP7 encodes G479, whilst in C87, it is R479. To examine whether this single amino acid exchange could explain differences in the ability of the viruses to infect the CD46 knock-out clones, we first verified the identity of the nucleotide 1819 of the viral genome—which determines the presence of G versus R at position 479 of the polyprotein—by RT-PCR and sequence analysis of the virus stocks employed for infection. The exchange G479R was present in C87, absent in NADL, and a mixed phenotype encoding either G or W was detected in CP7 (relevant sections of the sequencing chromatogram are shown in Supplementary Figure S1). Therefore, the presence of R479 correlated well with the observed ability to infect the CD46 knock-out clones.

To confirm that the observed reduced dependence on CD46 of C87 was indeed caused by the amino acid exchange G479R, we generated a C87 clone harboring a G at amino acid position 479. This clone was tested for susceptibility in comparison to the parental C87 with the CD46 knock-out clone 7 (Figure 1C), as this clone had exhibited the highest differences in susceptibility between C87 and NADL in previous experiments (Figure 1B). Susceptibility of the knock-out cell line indeed reduced from 59% to 7.8%, thereby nearly reaching the susceptibility to NADL (4.2%).

## 3. Discussion

The role of CD46 as a receptor for BVDV has already been established for more than a decade. Due to the lack of easy-to-use knock-out technology, the function of CD46 was for a long time primarily characterized by transcomplementing porcine cells with bovine CD46 and modifications thereof. Therefore, the importance of CD46 for BVDV entry in the background of bovine cells remained difficult to quantify. The recent generation of MDBK and SK6 CD46 knock-out cell lines was an important step to confirm the importance of CD46 in the entry of BVDV [13] and APPV [10]. Interestingly, the dependence on CD46 could easily be overcome by adaptions in the viral E^rns^ protein in the BVDV system [13]. One single amino acid exchange, already reported previously for CSFV to increase the affinity of virus particles for surface glycosaminoglycans [20], sufficed to rescue otherwise poorly growing BVDVs on CD46 knock-out cells [13].

Our results also demonstrated the dependence on CD46 if BVDVs do not encode the amino acid exchange G479R in E^rns^. By analyzing the susceptibility of the cell lines, we observed an approximately 20-fold reduced efficiency to infect cells if CD46 was non-functional. This reduction is substantial, but far from an on–off decisive factor for cellular susceptibility. Therefore, it is likely that more essential factors for BVDV entry must exist, which is also supported by the recent discovery of ADAM17 as an essential entry factor for CSFV [21]. The reduction of infected cells 24 h after infection by FACS analysis as performed by Szillat et al. [13] reported higher values for strain NADL. This divergence might be caused by our susceptibility assay not accounting for cell-to-cell spread or divergence in the cells or viruses employed in these studies.

CD46 as an entry factor is almost irrelevant in the presence of the G479R exchange, as the most susceptible knock-out clone had a susceptibility of 70% to C87. This high susceptibility was reduced if a C87 encoding G479 was employed in the experiment, to levels resembling the susceptibility for strain NADL. Whether the G479R exchange results in an attenuation in the natural host as observed for CSFV still needs to be evaluated [22]. Interestingly, overexpression of CD46 in porcine cells increased their susceptibility to C87 more than 80 times when compared to unmodified SK6 cells [16], demonstrating an importance of bovine CD46 even in the presence of G479R. This might indicate a lack or an incompatibility of yet unknown factors in porcine cells that allow for the highly efficient, CD46-independent infection of bovine cells in the presence of the G479R exchange.

The susceptibility of the different double knock-out clones varied substantially for C87, with an up to five times difference in susceptibility. This phenomenon was much less pronounced for CP7, with an up to 2.5 times variability in susceptibility between the different clones, and lowest for NADL with less than two times variability. Given these results, it is possible that the clonal differences are more pronounced when the knocked-out factor is not relevant for the virus. This clonal variability has however to be taken into consideration as the effect of CD46 knock-out on the susceptibility to NADL versus C87 would be estimated between 3.6 and 16.6 times increased depending on the clone employed. Therefore, our results confirm that off-target modifications and clonal selection affect the knock-out phenotype and suggest a correlation between the extent of these clonal effects and the dependence of the virus on the targeted factor.

Deletions in the modified clones that did not affect the reading frame confirmed the importance of the EQIV motive within CD46 for BVDV binding. The absence of this motive led to a susceptibility to NADL comparable to that of double knock-out clones, whilst the deletion of eight amino acids just prior to this motive reduced susceptibility to NADL less than three times. This rather weak reduction is surprising, as such a deletion should affect the localization and orientation of the EQIV harboring antiparallel beta sheet in the CD46 structure.

The cell clones encoding deletions instead of frame shifts in the CD46 gene also allowed us to assess the previously reported binding sites of antibodies CA17 and CA26. For CA26, we could confirm the binding to the CCP1 domain [11] and map amino acids important for binding to residues 62–74, indicating a direct interaction of the antibody with the BVDV E2 CD46 interaction site. The decreased signal intensity obtained with CA17 for two clones indicates that amino acids within the BVDV E2 binding site of CD46 are likely affecting its binding affinity. As this antibody’s binding site was previously mapped to CCP2 [11], it is currently unclear whether the antibody is interacting with amino acids in CCP1 or whether their deletion results in structural modifications that render antibody binding less efficient.

## 4. Materials and Methods

### 4.1. General Cell Culture

MDBK and HEK293 cells were kept in an incubator at 37 °C, 5% CO_2_, and 100% humidity in full medium consisting of DMEM 4.5 g/L glucose (Biowest, Nuaillé, France) supplemented with 100 U/mL penicillin and 100 µg/mL streptomycin and 10% FCS (Corning, Glendale, CA, USA). The FCS was tested BVDV-free through serial passaging and PCR analysis in our laboratory.

### 4.2. Generation of CD46 Knock-Out Cell Lines

Two guide RNAs (sgRNA, sgRNA(A): ATTGTGTATGAATGTCGTCTGGG; sgRNA(B): TCATACACAATCTGCTCCCCAGG) (Figure 1) were cloned into pLentiCRISPRv2 (Addgene 52961) according to the protocol by [23,24], yielding plasmids pH175 and pH176. Lentiviral pseudotypes were generated by transfecting 300 ng pH175 or pH176, 225 ng psPAX2, 150 ng pLP2, and 100 ng pVSVG into 1.5 × 10^5^ HEK293 cells seeded in a 24-well plate with polyethylenimine. Supernatant was harvested 48 h after transfection and precleared by spinning for 5 min at 13,000× *g* in a table-top centrifuge. An amount of 250 µL of pseudotype-containing supernatant was mixed with polybrene (8 µg/mL final concentration) and 2.5 × 10^4^ MDBK cells. After 4 h, medium was exchanged to DMEM containing 10% FCS. Forty-eight hours after transduction, cells were transferred to a 6-well plate and 1 µg/mL puromycin was added to the medium and replaced every 3–4 days. Seven days into the puromycin treatment, cells were detached by tryptic digestion and seeded at one cell/well in 96-well plates. For pH175 and pH176, eight 96-well plates each were employed. Once colonies were clearly visible in the wells, the cells were stained with the anti-CD46 antibody CA26 [12]. For this purpose, hybridoma supernatant of CA26 was diluted 1:100 in full medium and applied to the cells. After 45 min, the antibody was removed, and cells were washed three times with PBS. For detection, a goat anti-mouse Cy3-labelled polyclonal serum (Dianova, Hamburg, Germany) was diluted in full medium 1:300 and applied onto the plates. After an additional 45 min of incubation, cells were washed three times with PBS and full medium without phenol red was added. The CD46 staining was analyzed on an Olympus IX70 fluorescence microscope (OLYMPUS, Hamburg, Germany). When only one colony per well was present and this colony did not react with CA26, cells were detached by tryptic digestion and transferred to a new 96-well plate and serially diluted for clonal selection. In cases where several colonies were present in one well but only one was CD46 negative, this clone was picked with a 10 µL pipette tip and transferred to a new 96-well plate. After an additional staining, these clones were also subjected to further clonal selection if the CD46-negative phenotype could be confirmed.

Clonal selection was performed three times, and each time the lowest cell dilution was stained with antibodies blocking BVDV entry [12], CA26 (mapped to CCP1, [11]), and CA17 (mapped to CCP2, [11]) to verify the loss of CD46. The knock-out phenotype was again determined by staining with CA26 and CA17, this time after fixation with 4% PFA, and images were acquired with an XM10 Olympus camera mounted on an Olympus IX70 fluorescence microscope. Before image acquisition, cell nuclei were stained with DAPI 1 µg/mL for 5 min at RT. Images were acquired at 10-fold magnification, and exposure times were the same for all clones.

### 4.3. Genetic Confirmation of CD46 Knock-Out/Modification

The genotype of the knock-out clones was determined by a PCR specifically amplifying exon 2 (Figure 1A-i), black arrows indicate primer location). Genomic DNA (gDNA) was extracted from 1 × 10^6^ cells with the Monarch gDNA purification kit (NEB, Ipswich, MA, USA) according to the manufacturer’s instructions. PCR was performed with the primer pair R563 (GATGCTGTCTCTTCCATTTACT)/R564 (GCCTGAATGCATGGCTATCT) and the OneTaq (NEB) quick load 2× master mix. Specific bands were detected by gel electrophoresis, excised, and purified with the Monarch gel purification kit (NEB). The PCR product was sent for Sanger sequencing (Eurofins, Ebersberg, Germany), ligated into the pGEM-T Easy vector (Promega, Madison, WI, USA) and transformed into bacteria. DNA from clonal bacterial colonies was isolated and sent for sequencing. The sequence of the PCR product usually contained two sequence traces commencing at the sgRNA target site. By comparing the sequence of the PCR product with the single allele sequence derived from the bacterial clones with the Sanger sequence tracing program Indigo (https://www.gear-genomics.com/indigo/, accessed on 30 October 2021) [25], the genetic modification of the two alleles in MDBK cells was determined.

### 4.4. Determination of Susceptibility

MDBK cells (1 × 10^5^) or one subclone of each knock-out genotype were seeded into each well of a 24-well plate. After 24 h, cells were infected with a 10-fold serial dilution of the BVDV strains C87, CP7, or NADL. Four hours after virus addition, the medium was exchanged to full medium without phenol red containing 1% carboxymethylcellulose. Forty-eight hours after infection, medium was removed, and the cells were fixed with 4% paraformaldehyde for 20 min at 4 °C. After permeabilization with 1% Triton X100 for 5 min at RT, cells were washed with PBS 0.1% TWEEN20 (PBS-T) and incubated with the mouse monoclonal anti-BVDV-E2 antibody 6A5 [26] for 45 min at 37 °C. After three washing steps with PBS-T, a goat anti-mouse Cy3-labeled polyclonal serum (Dianova, Hamburg, Germany) was added to the cells and incubated for 45 min at 37 °C. The titer in FFU/mL was determined by counting the infected foci in wells containing between 1 and 100 foci by fluorescence microscopy after washing three times with PBS-T. The susceptibility in percent was calculated by dividing the titer in FFU/mL determined for each CD46 knock-out clone by the titer in FFU/mL determined for MDBK cells. Experiments were performed as biological triplicates.

### 4.5. Determination of Modifications in the E^rns^ Coding Region

Viral RNA was extracted from the virus stocks used in the infection experiments employing the QiaAMP viral RNA mini kit (Qiagen, Hilden, Germany) according to the manufacturer’s instructions. Subsequently, a one-step RT-PCR employing the primers Erns forward (NADL nt 1358–1379; CATGGTATGATGGATGCAAGTG) and E^rns^ reverse (NADL nt 2018–2040; GACAAGTGACCTCCCATCTCATG) was performed with the OneTaq One-Step RT-PCR kit (NEB) according to the manufacturer’s instructions. The resulting PCR product was purified with the Monarch PCR purification kit (NEB) according to the manufactures instructions and sent for sequencing (Eurofins) with the primer R588.

### 4.6. Statistical Analysis

Student’s *t*-test was employed to evaluate the statistical significance of the results.

## Data Availability

Not applicable.

## Supplementary Materials

The following are available online at https://www.mdpi.com/article/10.3390/pathogens10121620/s1, Figure S1: Interaction of MDBK CD46 knock-out clones with anti-BDVD antibodies CA26 and CA17; Figure S2: Presence of a G vs a R residue at amino acid position 479 in the different BVDV strains employed in this study.
