# Supplementary material for: Viral Traits and Cellular Knock-Out Genotype Affect Dependence of BVDV on Bovine CD46
Source: Pathogens. 2021 Dec 14;10(12):1620. doi: 10.3390/pathogens10121620 (PMC8704300; doi:10.3390/pathogens10121620)
Supplement: Supplementary file 1 [file pathogens-10-01620-s001.zip › pathogens-1491716-supplementary.pdf]

Supplementary material

Supplementary figure S1:

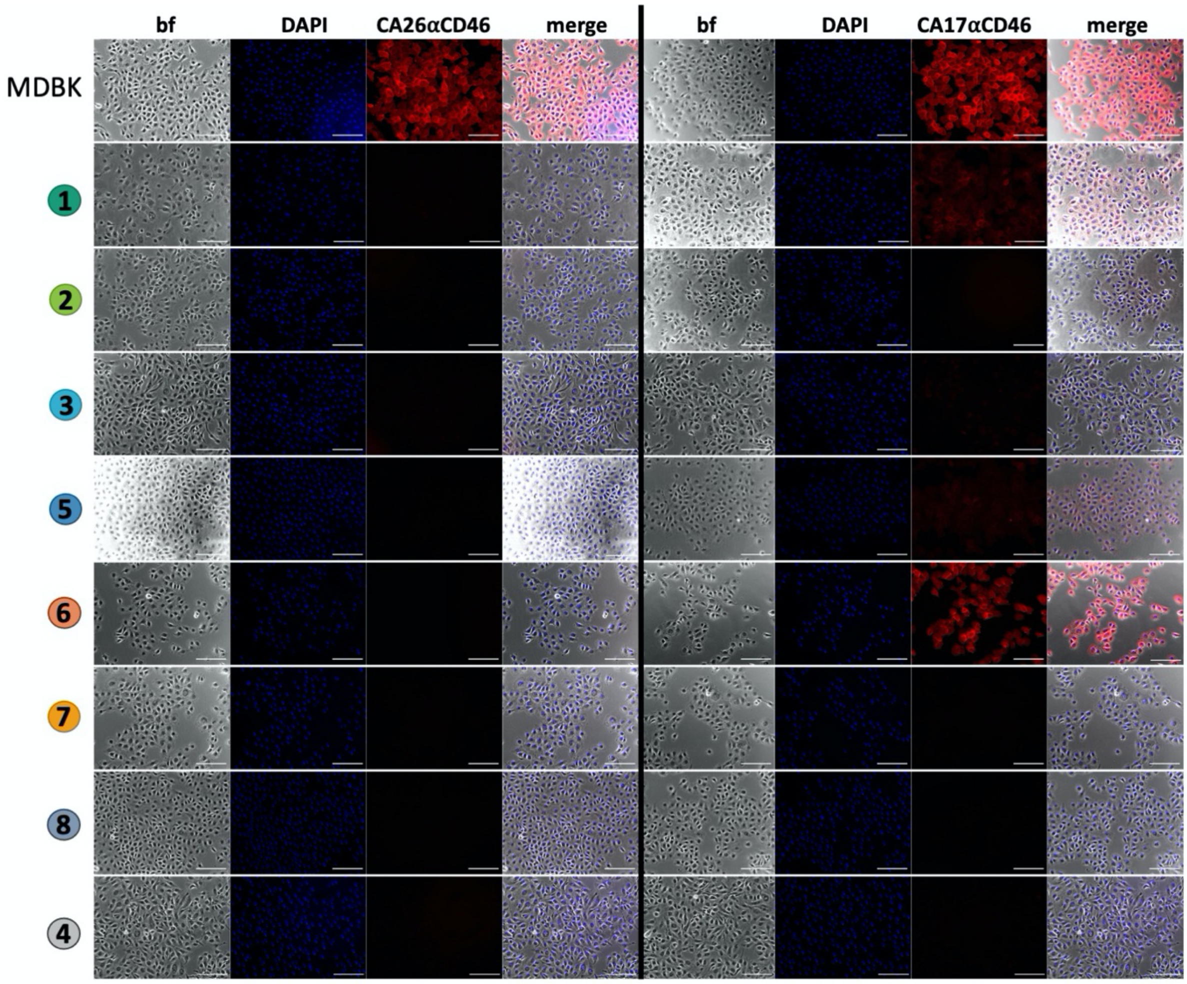

Interaction of MDBK CD46 knock-out clones with anti-BDVD antibodies CA26 and CA17. Cells are numbered according to figure 1. Shown are a brightfield image (bf), nuclear staining with DAPI (DAPI), staining with a CD46 interacting antibody (CA26αCD46 [left side] or CA17αCD46 [right side]) and an overlay of the different channels (merge). The scale bar represents 200µm.

Supplementary figure S2

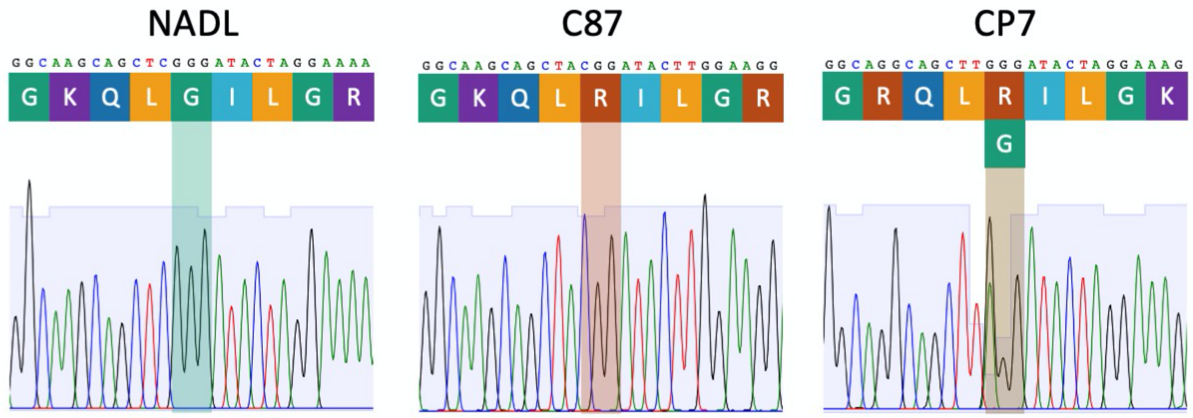

Presence of a G vs a R residue at amino acid position 479 in the different BVDV strains employed in this study. Shown are the relevant parts of the sequencing chromatogram of the E^{rns} coding region of the virus stocks employed in this study. Please note the nucleotide dimorphism in CP7.
